# Supplementary material for: ACEGEN: Reinforcement Learning of Generative Chemical Agents for Drug Discovery
Source: J Chem Inf Model. 2024 Aug 2;64(15):5900–11. doi: 10.1021/acs.jcim.4c00895 (PMC11581341; doi:10.1021/acs.jcim.4c00895)
Supplement: Supplementary file 2 — ci4c00895_si_002.pdf [file ci4c00895_si_002.pdf]

Supporting Information
ACEGEN: Reinforcement learning of generative chemical agents for drug discovery

Albert Bou,[†,‡] Morgan Thomas,[†] Sebastian Dittert,[†] Carles Navarro,[‡] Maciej Majewski,[‡] Ye Wang,[¶] Shivam Patel,[§] Gary Tresadern,[‖] Mazen Ahmad,[‖] Vincent Moens,[⊥] Woody Sherman,[§] Simone Sciabola,[¶] and Gianni De Fabritiis[*,#,†,‡]

†*Computational Science Laboratory, Universitat Pompeu Fabra, Barcelona Biomedical Research Park (PRBB), C Dr. Aiguader 88, 08003 Barcelona, Spain.*
‡*Acellera Labs, C Dr Trueta 183, 08005, Barcelona, Spain*
¶*Biogen Research and Development, 225 Binney Street, Cambridge, Massachusetts 02142, United States*
§*Psivant Therapeutics, 451 D Street, Boston, Massachusetts 02210, United States*
‖*In Silico Discovery, Janssen Research & Development, Janssen Pharmaceutica N. V., Turnhoutseweg 30, B-2340 Beerse, Belgium.*
⊥*PyTorch team, Meta, 11 – 21 Canal Reach, London, N1C 4DB, UK*
#*Institució Catalana de Recerca i Estudis Avançats (ICREA), Passeig Lluis Companys 23, 08010 Barcelona, Spain*

E-mail: g.defabritiis@gmail.com

A. Reinforcement Learning Environment

```python
from acegen.rl_env import TokenEnv
from acegen.vocabulary import Vocabulary
from torchrl.collectors import RandomPolicy

# Create a vocabulary from a list of characters
chars = ["START", "END", "(", ")", "1", "=", "C", "N", "O"]
chars_dict = {char: index for index, char in enumerate(chars)}
vocab = Vocabulary.create_from_dict(chars_dict, start_token="START", end_token="END")

# Create an environment from the vocabulary
env = TokenEnv(
    start_token=vocab.start_token_index,
    end_token=vocab.end_token_index,
    length_vocabulary=len(vocab),
    batch_size=4, # Number of trajectories to collect in parallel
)

# Create a random policy
policy = RandomPolicy(env.full_action_spec)

# Take an environment step
step_tensordict = env.step(policy(env.reset()))
print(step_tensordict)
```

```
# Output:

TensorDict(
    fields={
        action: Tensor(shape=torch.Size([4]), device=cpu, dtype=torch.int32,
        done: Tensor(shape=torch.Size([4, 1]), device=cpu, dtype=torch.bool),
        next: TensorDict(
            fields={
                done: Tensor(shape=torch.Size([4, 1]), device=cpu, dtype=torch.bool),
                observation: Tensor(shape=torch.Size([4]), device=cpu, dtype=torch.int64),
                reward: Tensor(shape=torch.Size([4, 1]), device=cpu, dtype=torch.float32),
                terminated: Tensor(shape=torch.Size([4, 1]), device=cpu, dtype=torch.bool),
                truncated: Tensor(shape=torch.Size([4, 1]), device=cpu, dtype=torch.bool)},
            batch_size=torch.Size([4]),
            device=cpu,
            is_shared=False),
        observation: Tensor(shape=torch.Size([4]), device=cpu, dtype=torch.int32),
        terminated: Tensor(shape=torch.Size([4, 1]), device=cpu, dtype=torch.bool),
        truncated: Tensor(shape=torch.Size([4, 1]), device=cpu, dtype=torch.bool)},
    batch_size=torch.Size([4]),
    device=cpu,
    is_shared=False)
```

Figure S1: Code example that shows how to create a language-based reinforcement learning environment components for SMILES generation and how to take sampling steps on it with a random policy.

B. Define Scoring Functions

A scoring function is simple a python function that takes a molecule (here in the form of SMILES) and returns a reward that describes the molecules desirability in relation to the objective (here in the region 0-1). Figure S2 is a demonstration of how to write a simple scoring function that evaluates the molecules QED[1] using RDKit. Whereas, Figure S3 demonstrates how MolScore[2] can be imported and used to configure scoring functions with a broad suite of available functionality. More detailed description of how to integrate these scoring functions into ACEGEN can be found on the GitHub in our tutorials section.

```python
from rdkit.Chem import AllChem, QED
from acegen import Task

def evaluate_mol(smiles: str):
    mol = AllChem.MolFromSmiles(smiles)
    if mol:
        return QED(mol)
    else:
        return 0.0

task = Task(
    name="custom_scoring_function",
    scoring_function=evaluate_mol,
    budget=10_000,
)

env =  TokenEnv(
    start_token=vocab.start_token_index,
    end_token=vocab.end_token_index,
    length_vocabulary=len(vocab),
    batch_size=1,
)

data = env.rollout(max_steps=100)
smiles_str = [vocab.decode(smi.numpy()) for smi in data["action"]]
reward = task(smiles_str)
```

Figure S2: An example of custom scoring function that returns a reward based on an input molecule. If the molecule is invalid, no reward is returned (i.e., 0.0).

```python
from molscore.manager import MolScore

task = MolScore(
    model_name="example",
    task_config=path/to/molscore/config/MolOpt/Albyterol_similarity.json,
    budget=10_000,
    output_dir="/tmp",
))

env =  TokenEnv(
    start_token=vocab.start_token_index,
    end_token=vocab.end_token_index,
    length_vocabulary=len(vocab),
    batch_size=1,
)

data = env.rollout(max_steps=100)
smiles_str = [vocab.decode(smi.numpy()) for smi in data["action"]]
reward = task(smiles_str)
```

Figure S3: An example scoring function created using MolScore.

C. Comparison against an existing implementation

We conducted a comparative analysis between our Reinvent implementation and MolOpt's[3] implementation, which is accessible on their Github repository. To ensure a fair comparison, we replaced only the prior weights and utilized MolScore for computing scoring functions. Apart from these adjustments, we maintained the use of the original code. Both implementations use the same network architecture and hyperparameters.

Table S1 and Table S2 show that our code is faster and obtains higher values for all metrics with compared to that implementation. The observed performance gaps may stem from subtle idiosyncrasies within the codebase. In reinforcement learning, even minor variations can yield notable discrepancies in outcomes. This underscores the importance of employing standardized, rigorously tested components and foundational building blocks, which offer heightened reliability. Furthermore, we hypothesize the enhanced efficiency can be attributed to leveraging components from TorchRL, a part of the META ecosystem, which inherently provides better optimization and reliability compared to bespoke solutions.

Table S1: Comparison of REINVENT MolOpt Implementation and REINVENT ACEGEN Implementation code on a machine with 32 CPUs and a NVIDIA GeForce RTX 4090 GPU for different scoring functions. In each run the algorithm generates and trains on 10K molecules.

Task	REINVENT MolOpt Implementation	REINVENT ACEGEN Implementation
Albuterol similarity	2:06 mins	**1:01 mins**
Amlodipine MPO	5:05 mins	**3:25 mins**
C7H8N2O2	1:44 mins	**0:42 mins**
C9H10N2O2PF2Cl	1:44 mins	**0:37 mins**

Table S2: Comparison of REINVENT MolOpt Implementation and ACEGEN AceGen Implementation on the MolOpt benchmark scoring functions. The results are presented as the sum of all scoring function in the benchmark for different metrics. Each algorithm ran 5 times with different seeds, and results were averaged.

Metric	REINVENT MolOpt Implementation	REINVENT ACEGEN Implementation
Avg top 10	15.833	**15.959**
Avg top 100	14.640	**14.896**
AUC top 10	13.597	**13.740**
AUC top 100	11.873	**11.985**

D. MolOpt Benchmark

The MolOpt benchmark was run using the MolScore implementation for convenience as it automatically calculates the same metrics as in the original implementation. In addition, MolScore computes the metrics after the removal of undesirable chemistry via the use of a basic chemistry filter (B-CF). This filter ensures molecules have a logP less than or equal to 4.5, rotatable bond count less than or equal to 7, molecular weight in the range 150 to 650 Da, only contain atoms belonging to the following set $A \in \{C, S, O, N, H, F, Cl, Br\}$, and do not violate the substructure alerts as described in.[4] If provided with a set of reference molecules, MolScore computes the metrics after the remove of undesirable chemistry via the use of a target chemistry filter (T-CF). This filter ensures molecules have a molecular weight and logP range within $\mu \pm 4\sigma$ of the reference distribution, as well as, removing any molecule that comprises $> 10\%$ novel atomic environment bits with respect to the reference molecules, as measured by ECFP4 bits (an example of this is shown in Figure S6). Lastly, MolScore also computes the metrics after filtering molecules using both basic and target chemistry filters (B&T-CF).

Table S3: Performance summary of algorithms for further metrics measured in this benchmark. Each algorithm ran 5 times with different seeds, and results were averaged and then summed over tasks.

Metric	AceGen REINFORCE	AceGen REINVENT	AceGen REINVENT-MolOpt	AceGen AHC	AceGen A2C	AceGen PPO	AceGen PPOD
Valid	21.77	**21.78**	21.74	21.45	18.93	**21.78**	21.52
Top-1 Avg	16.60	16.41	**17.73**	16.77	16.35	16.14	17.47
Top-10 Avg	15.85	15.69	**17.43**	16.09	15.67	15.63	17.10
Top-100 Avg	14.59	14.41	**16.90**	14.95	14.66	14.81	16.50
Top-1 AUC	14.85	14.79	16.28	15.06	15.21	15.39	**16.41**
Top-10 AUC	13.67	13.60	15.65	13.91	14.27	14.65	**15.80**
Top-100 AUC	11.96	11.84	14.58	12.17	12.83	13.41	**14.67**
Top-1 Avg (Div)	16.60	16.41	**17.73**	16.77	16.35	16.14	17.47
Top-10 Avg (Div)	15.63	15.46	**17.13**	15.90	15.42	15.35	16.70
Top-100 Avg (Div)	13.89	13.76	**14.62**	14.21	13.68	12.63	13.54
Top-1 AUC (Div)	14.85	14.79	16.28	15.06	15.21	15.39	**16.41**
Top-10 AUC (Div)	13.53	13.47	**15.42**	13.76	14.09	14.37	15.39
Top-100 AUC (Div)	11.66	11.56	**13.07**	11.80	12.26	11.67	12.36
Unique	22.28	22.63	13.68	**22.68**	18.38	9.47	10.21
B&T-CF	14.34	**14.74**	7.00	13.77	7.82	5.81	5.71
B&T-CF Top-1 Avg	15.97	15.86	**16.85**	16.13	15.61	15.52	16.69
B&T-CF Top-10 Avg	15.21	15.07	**16.44**	15.44	14.83	14.99	16.32
B&T-CF Top-100 Avg	13.75	13.68	**15.61**	14.18	13.54	13.98	15.46
B&T-CF Top-1 AUC	14.26	14.20	15.60	14.47	14.54	14.81	**15.74**
B&T-CF Top-10 AUC	13.06	12.98	14.91	13.27	13.52	14.02	**15.11**
B&T-CF Top-100 AUC	11.24	11.15	13.61	11.44	11.88	12.64	**13.76**
B&T-CF Top-1 Avg (Div)	15.97	15.86	**16.85**	16.13	15.61	15.52	16.69
B&T-CF Top-10 Avg (Div)	14.95	14.83	**16.06**	15.25	14.54	14.60	15.78
B&T-CF Top-100 Avg (Div)	12.96	12.95	13.12	**13.31**	12.44	11.41	12.14
B&T-CF Top-1 AUC (Div)	14.26	14.20	15.60	14.47	14.54	14.81	**15.74**
B&T-CF Top-10 AUC (Div)	12.91	12.85	**14.61**	13.11	13.32	13.67	14.60
B&T-CF Top-100 AUC (Div)	10.91	10.84	**11.84**	11.04	11.21	10.59	11.19
B&T-CF Diversity (SEDiv@1k)	17.39	**18.19**	10.10	17.55	14.24	8.47	7.96

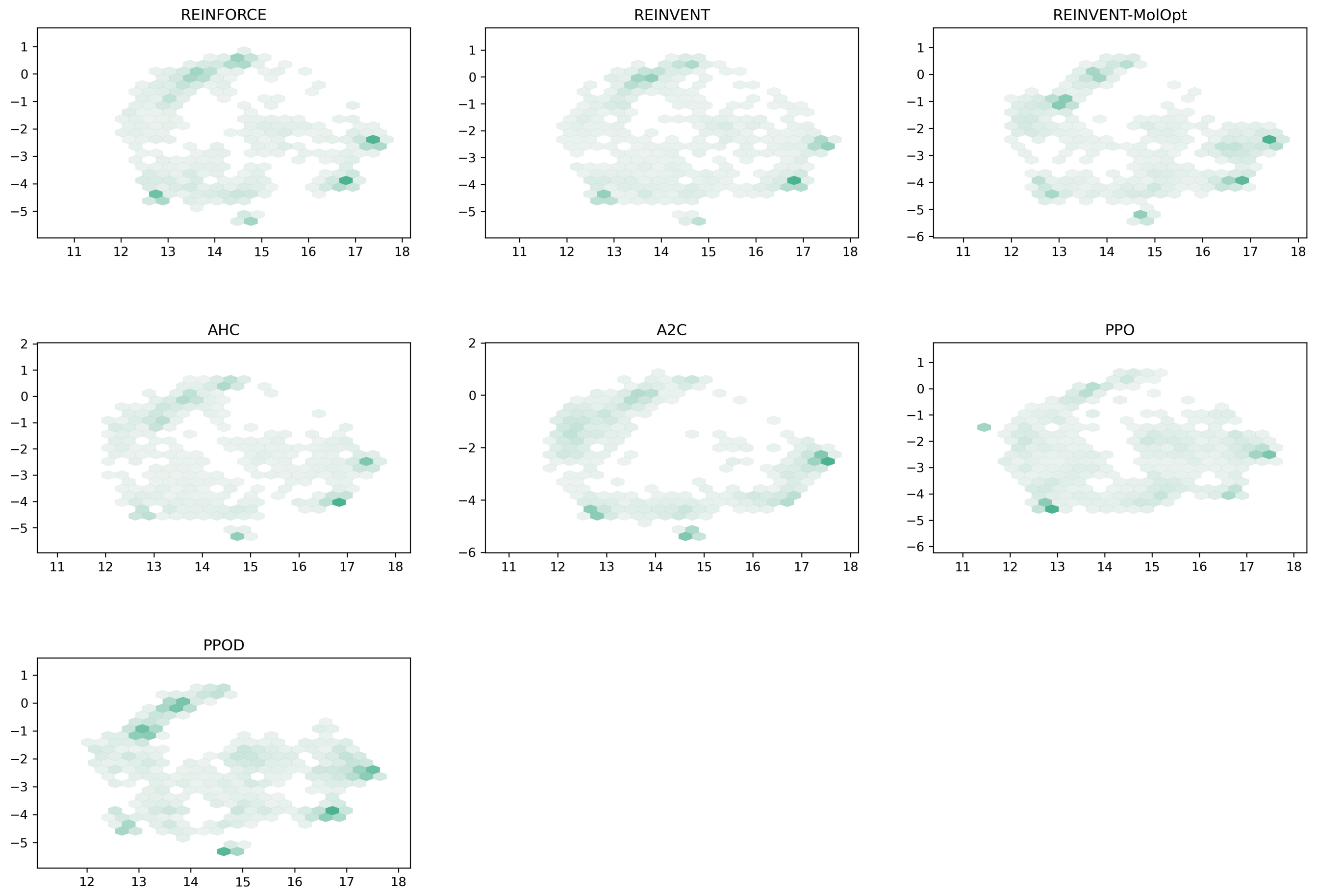

Figure S4: Visualization of the chemical space of the top 100 molecules across all tasks and repetitions by RL algorithm.

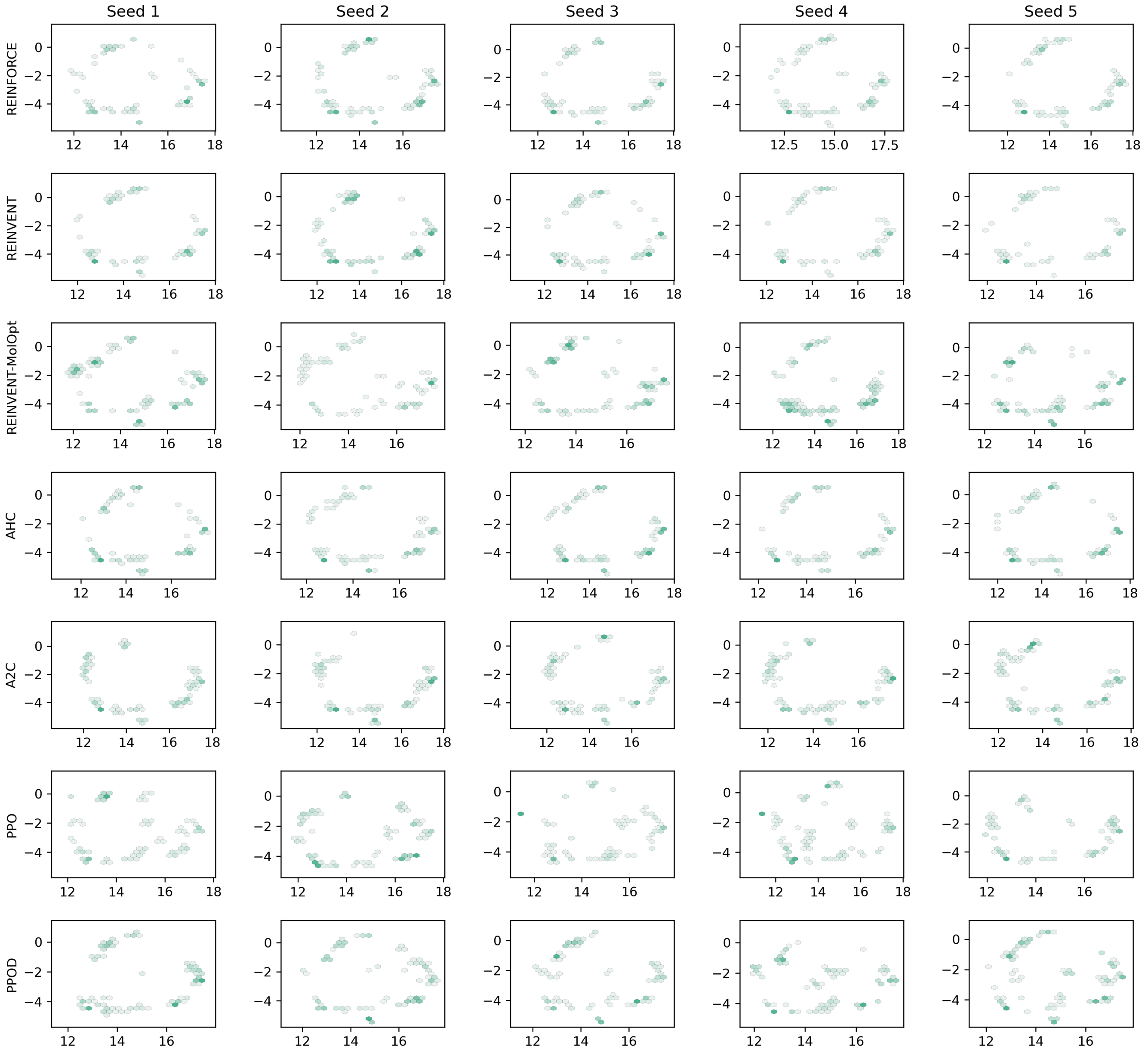

Figure S5: Visualization of the consistency of chemical space of the top 100 molecules across all tasks by repetition and RL algorithm.

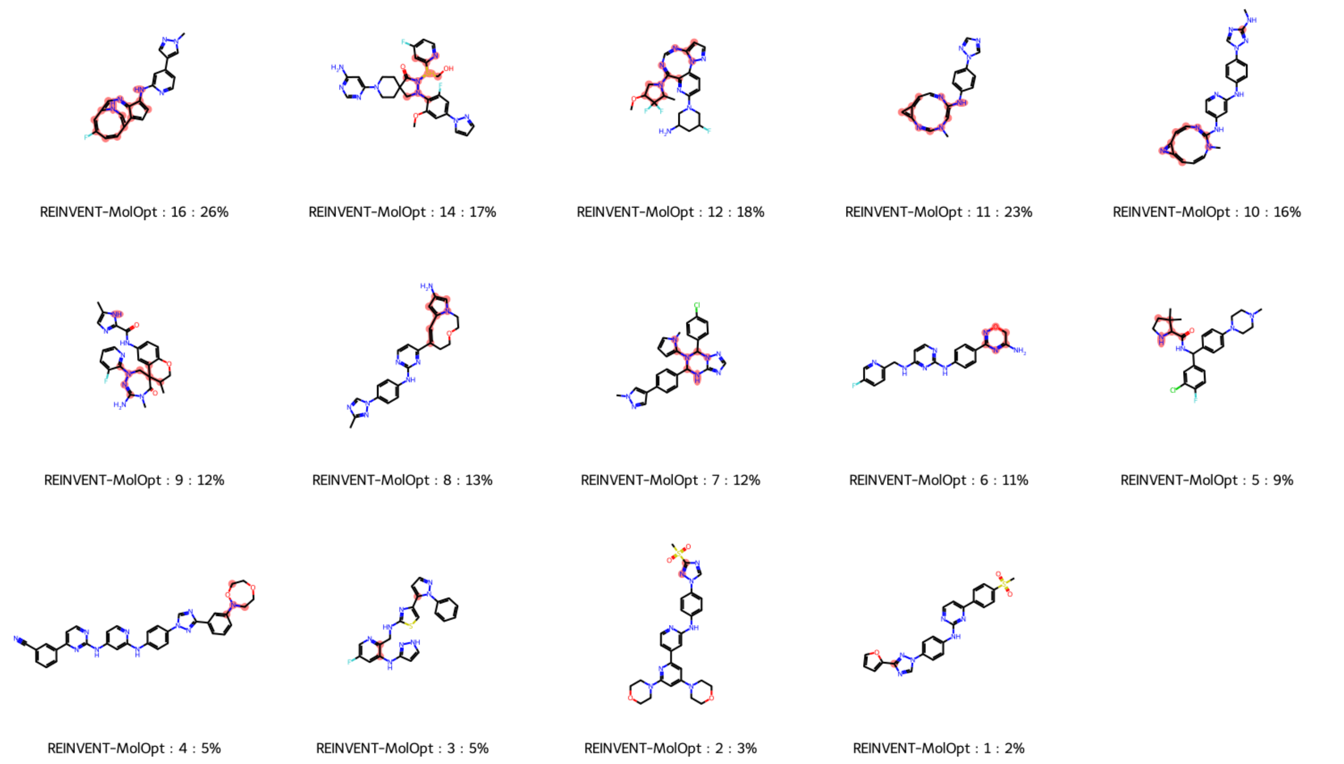

Figure S6: Demonstration of molecules measured as idiosyncratic by their percentage of novel ECFP4 bits relative to a reference dataset, with novel bits highlighted in red. Molecules are taken from those generated on the JNK3 task and are labelled by RL algorithm, number of novel bits, and percentage of novel bits relative to all bits present in the molecule.

E. 5-HT$_{2A}$ case study

The same training dataset and pre-trained prior was used as for the MolOpt benchmark. For the docking objectives, PDB structures 6A93[5] and 6CM4[6] were used for 5-HT$_{2A}$ the D_2 respectively. For further details of docking protocol see MolScore[?] and a configuration file to reproduce the objective can be found at https://github.com/MorganCThomas/MolScore/. The score for each molecule is in the range [0-1] and is calculated as a weighted sum of the individual scoring components described in Table S4. Note that during training, the inter-molecular rDock score was used as it was otherwise observed that RL would hack score optimization by improvement of intra-molecular rDock score only (data not shown). To discourage excessive exploitation, a diversity filter was used to penalize the re-use scaffolds similar to those previously sampled. The 126 known 5-HT$_{2A}$ ligands were extracted from ChEMBL31 and contained a pChEMBL$_{5-HT_{2A}}$ bioactivity value ≥ 6 and displayed atleast 100-fold selectivity versus D_2 (pChEMBL$_{5-HT_{2A}}$ − pChEMBL$_{D_2}$ ≥ 2). All metrics calculated during analysis were calculated using MolScore. Uniform manifold approximation and projection (UMAP)[7] was used to visualize chemical space, calculated using Jaccard distance with a minimum similarity of 0.1 and number of neighbours of 100 on ECFP6 fingerprints. The UMAP was fit to a random subset of 10,000 molecules taken from the prior training dataset. Normalized principal moments[8] were calculated using RDKit on the docked poses to visualize molecular geometry. Protein-ligand interaction fingerprints were calculated on docked poses using the ProLIF library.[9]

To further probe the differences in the chemistry proposed by the different RL algorithms, the metrics were calculated on all generated *de novo* molecules within the budget, shown in Tables S6, S7, and S8. Notably, ACEGEN-REINVENT-MolOpt achieved the best average 100 score but performed poorly with respect to chemistry-related metrics such as the ratio of molecules passing quality filters, chemical diversity and idiosyncratic atomic environments as measured by outlier bits with respect to the pre-training dataset. Other algorithms such as, ACEGEN-REINVENT with default parameters, AHC, PPO, and PPOD perform better in these chemistry-related metrics, suggesting an overall higher quality of chemistry proposed. This could reflect stronger regularisation towards the prior distribution, confirmed by higher similarity to the initial pre-training dataset (Table S7). In comparison to the extracted set of known 5-HT$_{2A}$ ligands (Table S8), ACEGEN-AHC manages to generate at least one analog to 78% of known ligands, followed by ACEGEN-REINVENT at 63% and then PPOD at 55%. A visualization of the chemical space explored is shown in S8, showing that ACEGEN-REINFORCE and ACEGEN-REINVENT are most explorative, followed by ACEGEN-AHC, ACEGEN-A2C, ACEGEN-PPO, REINVENT-MolOpt and finally ACEGEN-PPOD least exploratorive. This results in complementary chemical space occupation of the top 100 solutions by either algorithm (Figure S9), all of which show areas of overlap with the known 5-HT$_{2A}$ ligands. Interestingly, a number of the example molecules (Figure S10) contain caged sub-structures or macrocycles typically observed more frequently in natural products than typical small-molecule drugs. This could be a result of the selectivity required in the objective, where more 3D spherical structures are typically more specific binders, but are more challenging synthetically. This can also be seen by a slight shift towards spherical geometries by the ACEGEN-A2C and ACEGEN-PPOD compounds compared to the known selective ligands, shown in Figure S11.

A comparison of the protein interactions made by the top 10 compounds highlight the targeting of potentially selectivity inducing interactions. Figure S12 shows that many of the top 10 compounds proposed by ACEGEN-REINFORCE and ACEGEN-AHC form interactions with S131, T134, W151, and I152 in the second extended binding pocket which differ to those found in D_2, and therefore, could potentially be a site to obtain selectivity. For example, occupied by the caged substructure shown in the selected examples in Figure **??** proposed by ACEGEN-AHC, occupying a sub-pocket that is not sterically possible in the D_2 crystal structure used here. However, other D_2

structures do show occupation of this after re-arrangement of extracellular loop 1.[10] This reinforces the difficulty in identifying robust, validated scoring functions or oracles in drug discovery. For example, the use of Gypsum-DL[11] to prepare ligands for docking in this protocol leads to the generation of some implausible tautomers and unlikely protonation states and geometries (for example, cis-amides), meanwhile the docking parameters need further refinement to improve the quality of binding poses and cationic interactions with $D155^{3x32}$ which are sometimes in an unlikely orientation. These can be swapped for commercial products like LigPrep in MolScore provided the licence is obtained. However, this challenging objective enables the differentiation of RL performance and behaviour in the optimization of a specified objective and sets a realistic and practical benchmark for drug discovery.

Table S4: Description of the scoring functions to define the 5-HT_{2A} selective objective.

Scoring function	Comments	Target	Weight
5-HT_{2A} docking score (inter-molecular only)	- Positional cationic restraint for D^{3x32}<br>- Best score of any protonated/tautomerised/isomeric variant<br>- Normalized based on maximum/minimum values observed at timestep	Minimize	1
D_2 docking score (inter-molecular only)	- Best score of any protonated/tautomerised/isomeric variant<br>- Normalized based on maximum/minimum values observed at timestep	Maximize	0.5
Net positive charge		1	1
Maximum number of consecutive rotatable bonds		≤ 3	1

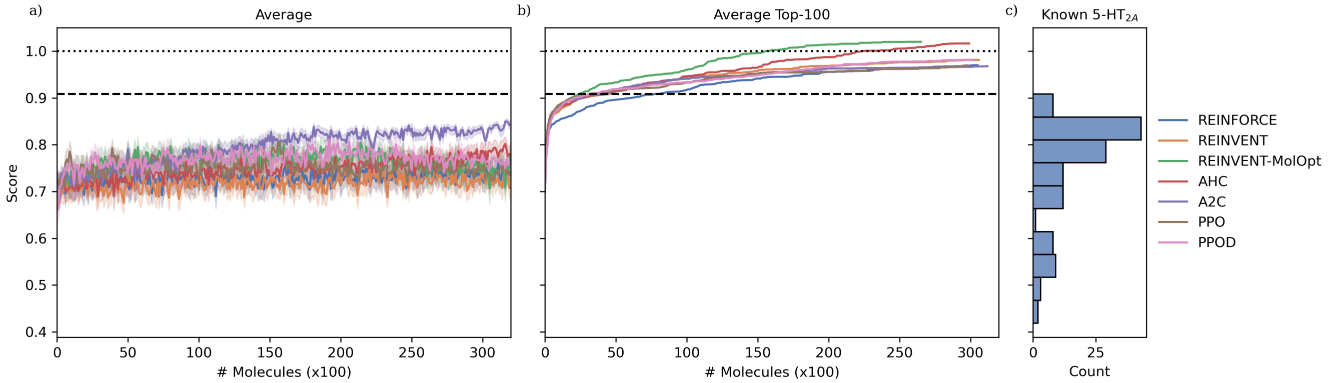

Figure S7: Optimisation of a challenging 5-HT_{2A} structure-based selectivity objective by different RL algorithms. The average score does not improve much over the course of training, but the average score of the top 100 compounds improves beyond known selective 5-HT_{2A} ligands. Note that the values here are re-normalized based on the scores achieved for a subset of 5-HT_{2A} ligands that display at least 2-fold selectivity over D_2 as extracted from ChEMBL31.[12] Therefore, a score of 0.91 indicates the best score observed in the known ligand subset, and a score greater than 1.0 indicates that a single molecule achieves both a better (more negative) 5-HT_{2A} docking score and (more positive) D_2 docking score than seen anywhere in the known subset.

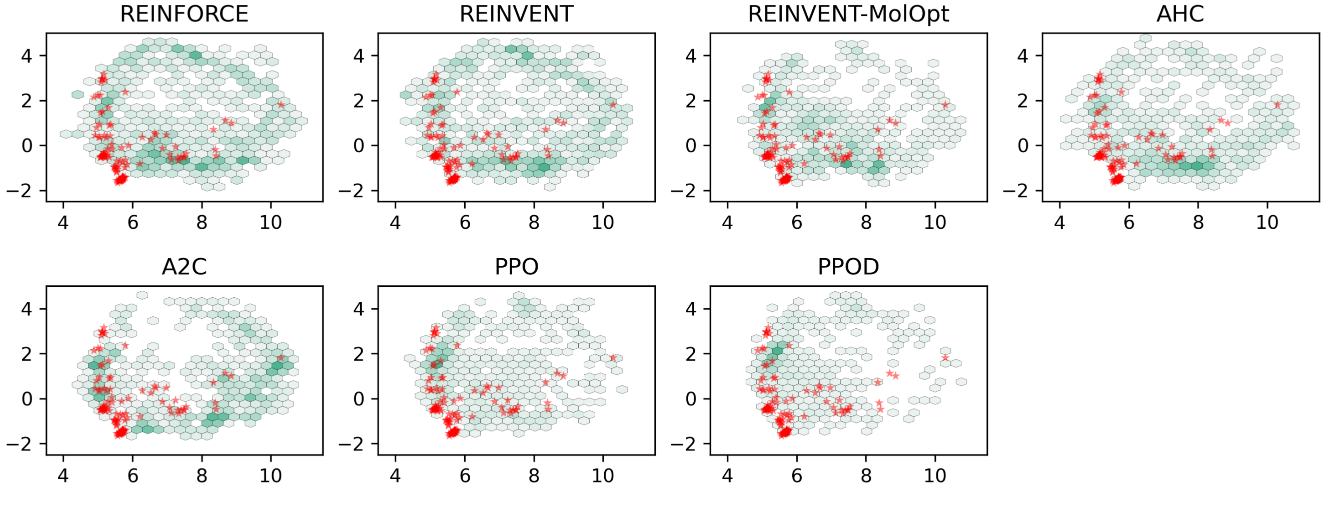

Figure S8: Chemical space explored by different RL algorithms as visualized by UMAP embedding. Known 5-HT_{2A} ligands are plotted in red.

Table S5: Performance summary of algorithms for average top-n metrics on 5-HT$_{2A}$ case study. Including after applying a basic chemistry filter (B-CF) as described previously. B-CF is the fraction of molecules that pass the chemistry filter.

Metric	ACEGEN REINFORCE	ACEGEN REINVENT	ACEGEN REINVENT-MolOpt	ACEGEN AHC	ACEGEN A2C	ACEGEN PPO	ACEGEN PPOD
Valid	0.95	0.96	0.93	0.94	**0.98**	0.94	0.95
Top-1 Avg	1.14	1.15	**1.20**	1.15	1.11	1.09	1.10
Top-10 Avg	1.05	1.09	**1.10**	**1.10**	1.04	1.06	1.07
Top-100 Avg	0.97	0.98	**1.02**	**1.02**	0.97	0.97	0.98
Top-1 AUC	1.10	**1.12**	**1.12**	**1.12**	1.09	1.06	1.08
Top-10 AUC	1.02	**1.05**	**1.05**	**1.05**	1.02	1.01	1.02
Top-100 AUC	0.93	0.95	**0.97**	0.96	0.94	0.94	0.94
Top-1 Avg (Div)	1.14	1.15	**1.20**	1.15	1.11	1.09	1.10
Top-10 Avg (Div)	1.05	1.08	**1.10**	**1.10**	1.04	1.06	1.07
Top-100 Avg (Div)	0.97	0.98	1.01	**1.02**	0.97	0.97	0.98
Top-1 AUC (Div)	1.10	**1.12**	**1.12**	**1.12**	1.09	1.06	1.08
Top-10 AUC (Div)	1.02	**1.05**	**1.05**	**1.05**	1.02	1.01	1.02
Top-100 AUC (Div)	0.93	0.95	**0.96**	**0.96**	0.94	0.93	0.94
Unique	**1.00**	**1.00**	0.89	**1.00**	**1.00**	0.99	0.99
B&T-CF	**0.71**	**0.71**	0.45	0.63	0.27	0.59	0.48
B&T-CF Top-1 Avg	1.14	1.13	1.12	**1.15**	1.11	1.08	1.10
B&T-CF Top-10 Avg	1.05	1.07	1.08	**1.10**	1.02	1.02	1.03
B&T-CF Top-100 Avg	0.96	0.97	**1.00**	**1.00**	0.93	0.95	0.96
B&T-CF Top-1 AUC	1.10	1.11	1.08	**1.12**	1.09	1.03	1.08
B&T-CF Top-10 AUC	1.01	1.03	1.04	**1.05**	1.01	0.99	0.99
B&T-CF Top-100 AUC	0.92	0.94	**0.96**	0.95	0.92	0.93	0.93
B&T-CF Top-1 Avg (Div)	1.14	1.13	1.12	**1.15**	1.11	1.08	1.10
B&T-CF Top-10 Avg (Div)	1.05	1.06	1.08	**1.10**	1.02	1.02	1.03
B&T-CF Top-100 Avg (Div)	0.95	0.97	0.98	**1.00**	0.93	0.95	0.95
B&T-CF Top-1 AUC (Div)	1.10	1.11	1.08	**1.12**	1.09	1.03	1.08
B&T-CF Top-10 AUC (Div)	1.01	1.03	1.04	**1.05**	1.00	0.99	0.99
B&T-CF Top-100 AUC (Div)	0.92	0.93	0.94	**0.95**	0.92	0.92	0.92
B&T-CF Diversity (SEDiv@1k)	0.89	**0.91**	0.64	0.89	0.77	0.60	0.53

Table S6: Summary of metrics measuring intrinsic properties of the chemistry generated by different algorithms. Most metrics are different measures of chemical diversity, therefore, higher is generally better but can sometimes be a result of idiosyncratic chemistry.

Metric	ACEGEN REINFORCE	ACEGEN REINVENT	ACEGEN REINVENT-MolOpt	ACEGEN AHC	ACEGEN A2C	ACEGEN PPO	ACEGEN PPOD
Validity	0.95	0.96	0.93	0.94	**0.98**	0.94	0.95
Uniqueness	**1.00**	**1.00**	0.89	**1.00**	**1.00**	0.99	0.99
Scaffold Uniqueness	0.91	0.90	0.76	0.95	**0.98**	0.80	0.84
Novelty	**1.00**	0.99	**1.00**	**1.00**	**1.00**	**1.00**	**1.00**
IntDiv	**0.87**	**0.87**	0.85	0.86	0.86	0.85	0.83
ScaffDiv	**0.85**	**0.85**	0.82	**0.85**	**0.85**	0.82	0.81
SEDiv@1k	0.89	**0.93**	0.64	0.92	0.58	0.70	0.61
SPDiv@1k	**0.89**	**0.89**	0.87	**0.89**	0.87	0.87	0.86
FG	0.02	0.02	0.01	0.03	0.01	0.04	**0.05**
RS	0.04	0.04	0.03	**0.05**	0.04	0.02	0.02
Quality Filters	**0.85**	0.84	0.70	0.84	0.56	0.84	0.78
Predicted Purchasability	0.01	**0.02**	0.01	0.01	0.01	**0.02**	0.01

Table S7: Summary of metrics measuring extrinsic properties of the chemistry generated by different algorithms with respect to the initial training dataset. Metrics are different measures of chemical similarity, therefore, higher is generally better (with the exception of OutlierBits) depending on how much it is desirable to regularize the model and maintain initial prior biases.

Metric	ACEGEN REINFORCE	ACEGEN REINVENT	ACEGEN REINVENT-MolOpt	ACEGEN AHC	ACEGEN A2C	ACEGEN PPO	ACEGEN PPOD
Analogue Similarity	**0.28**	0.27	0.09	0.21	0.05	0.21	0.22
Analogue Coverage	0.52	**0.54**	0.21	0.44	0.23	0.27	0.25
FG	0.96	**0.98**	0.68	0.91	0.69	0.90	0.66
RS	**1.00**	**1.00**	0.96	0.98	0.46	0.97	0.97
SNN	**0.37**	**0.37**	0.33	0.36	0.27	0.36	0.36
Frag	0.97	**0.98**	0.89	0.94	0.42	0.96	0.90
Scaf	0.30	**0.34**	0.07	0.19	0.07	0.14	0.11
OutlierBits	**0.08**	**0.08**	0.12	0.10	0.21	0.11	0.12

Table S8: Summary of metrics measuring extrinsic properties of the chemistry generated by different algorithms with respect to known 5-HT_{2A} ligands. Metrics are different measures of chemical similarity, therefore, higher is generally better (with the exception of OutlierBits) depending on how much it is desirable to rediscover known chemistry.

Metric	ACEGEN REINFORCE	ACEGEN REINVENT	ACEGEN REINVENT-MolOpt	ACEGEN AHC	ACEGEN A2C	ACEGEN PPO	ACEGEN PPOD
Analogue Similarity	**0.01**	0.00	0.00	0.00	0.00	0.00	**0.01**
Analogue Coverage	0.64	0.63	0.39	**0.78**	0.13	0.25	0.55
FG	**0.66**	0.65	0.59	0.65	0.42	0.62	0.59
RS	0.75	0.74	0.72	0.73	0.30	0.71	**0.76**
SNN	0.23	0.22	0.23	0.22	0.18	**0.24**	**0.24**
Frag	0.52	0.51	**0.59**	0.50	0.10	0.50	0.46
Scaf	**0.00**	**0.00**	**0.00**	**0.00**	**0.00**	**0.00**	**0.00**
OutlierBits	0.41	0.42	0.40	0.43	0.52	**0.38**	0.39

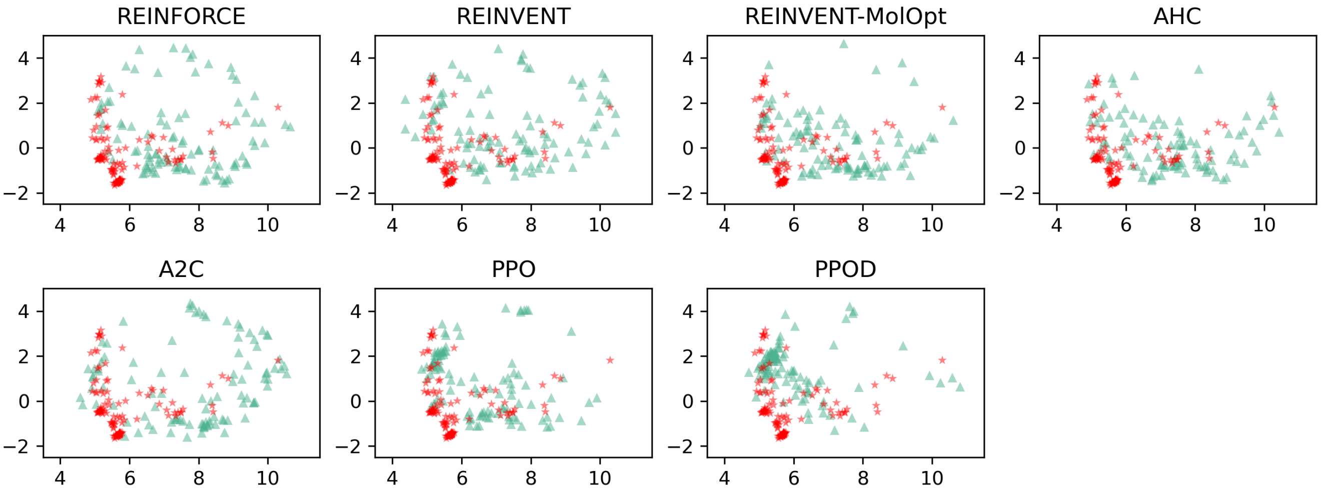

Figure S9: Chemical space of the top 100 molecules identified by each RL algorithms as visualized by UMAP embedding. Known 5-HT2A ligands are plotted in red.

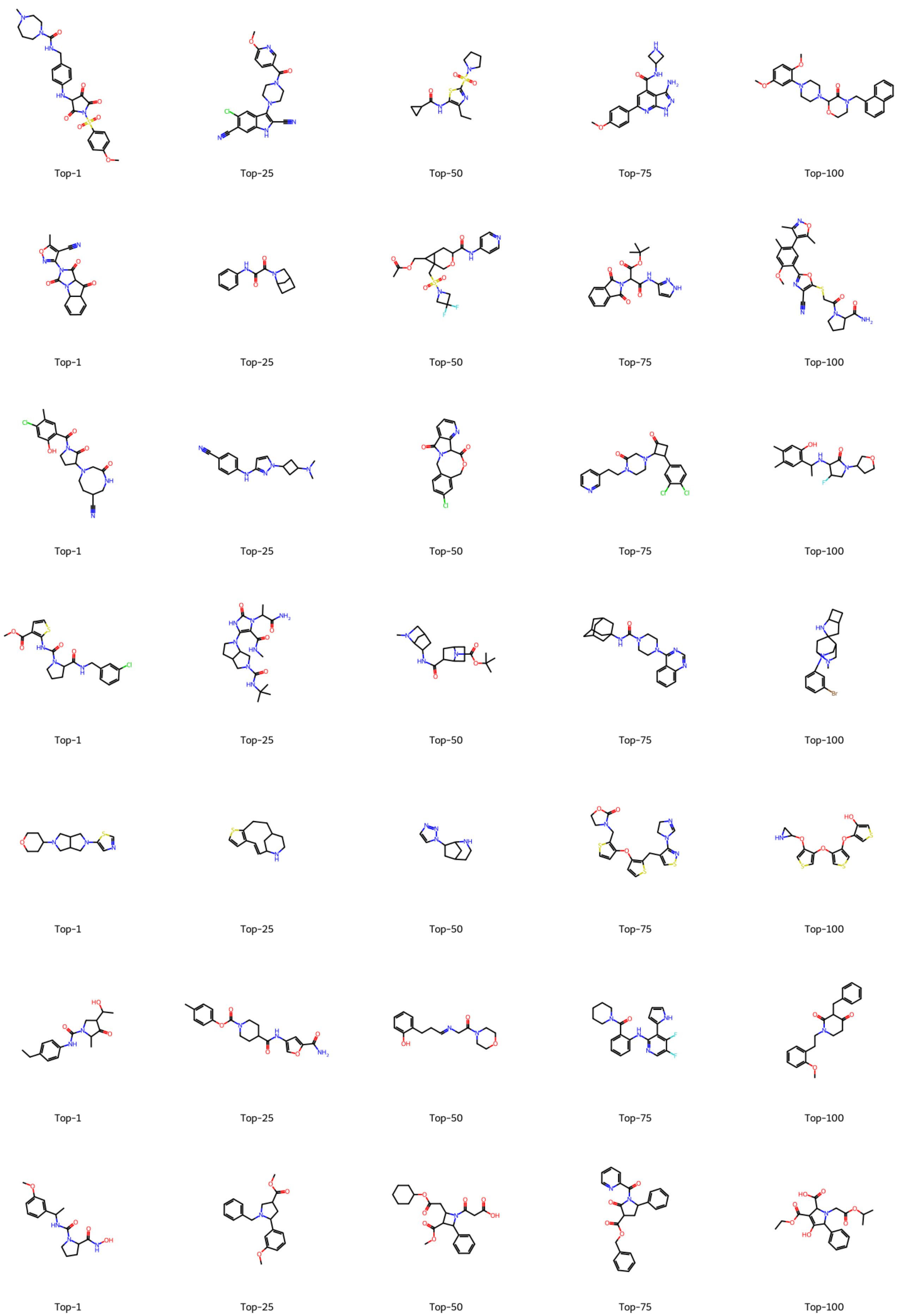

Figure S10: Five example molecules in the range of the top 100 by score. Each row represents REINFORCE, REINVENT, REINVENT-MolOpt, AHC, A2C, PPO and PPOD respectively.

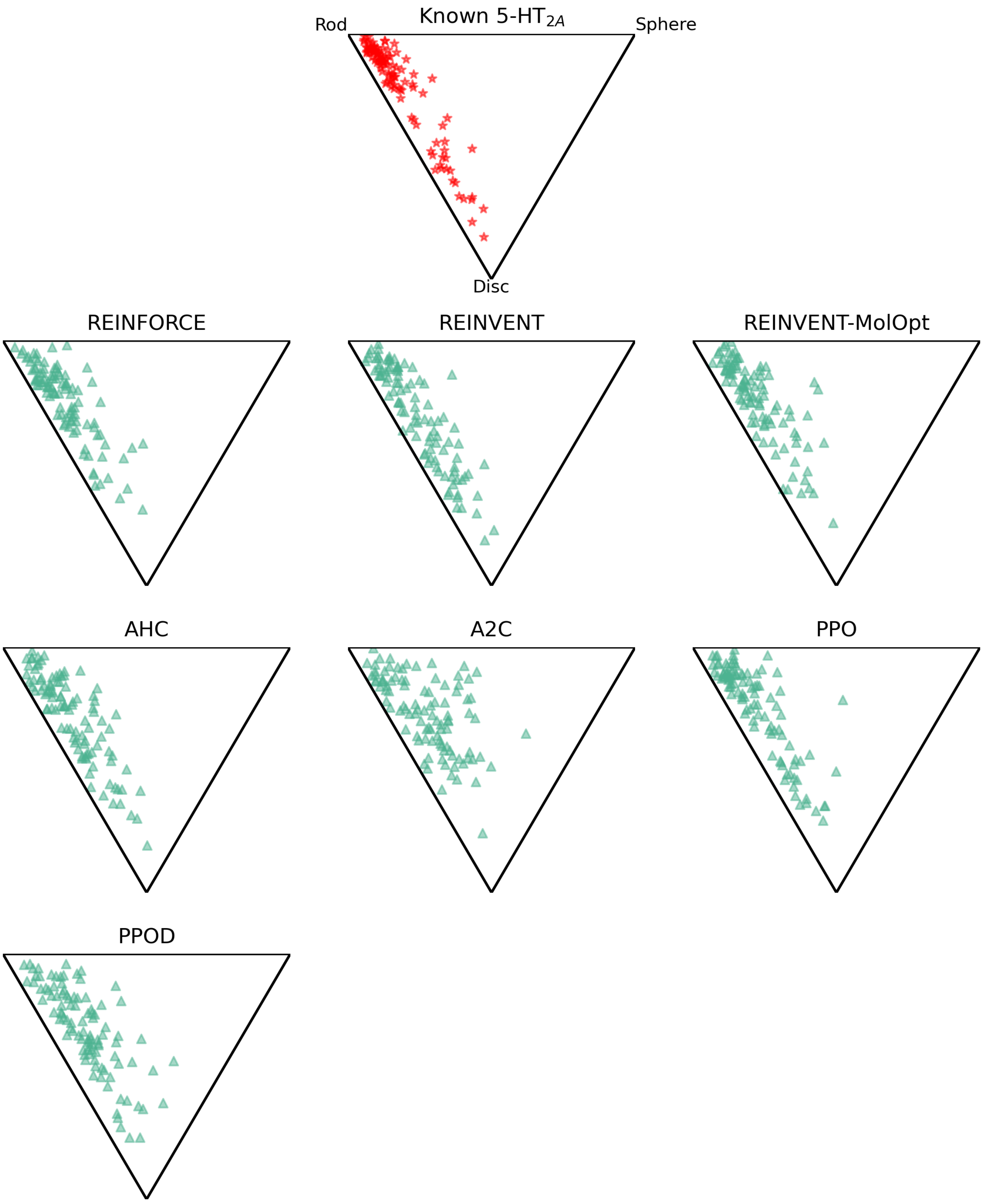

Figure S11: Normalized principal moment ratio space measuring the 3D geometrical shape of the known 5-HT$_{2A}$ ligands and top 100 from generated by the RL algorithms.

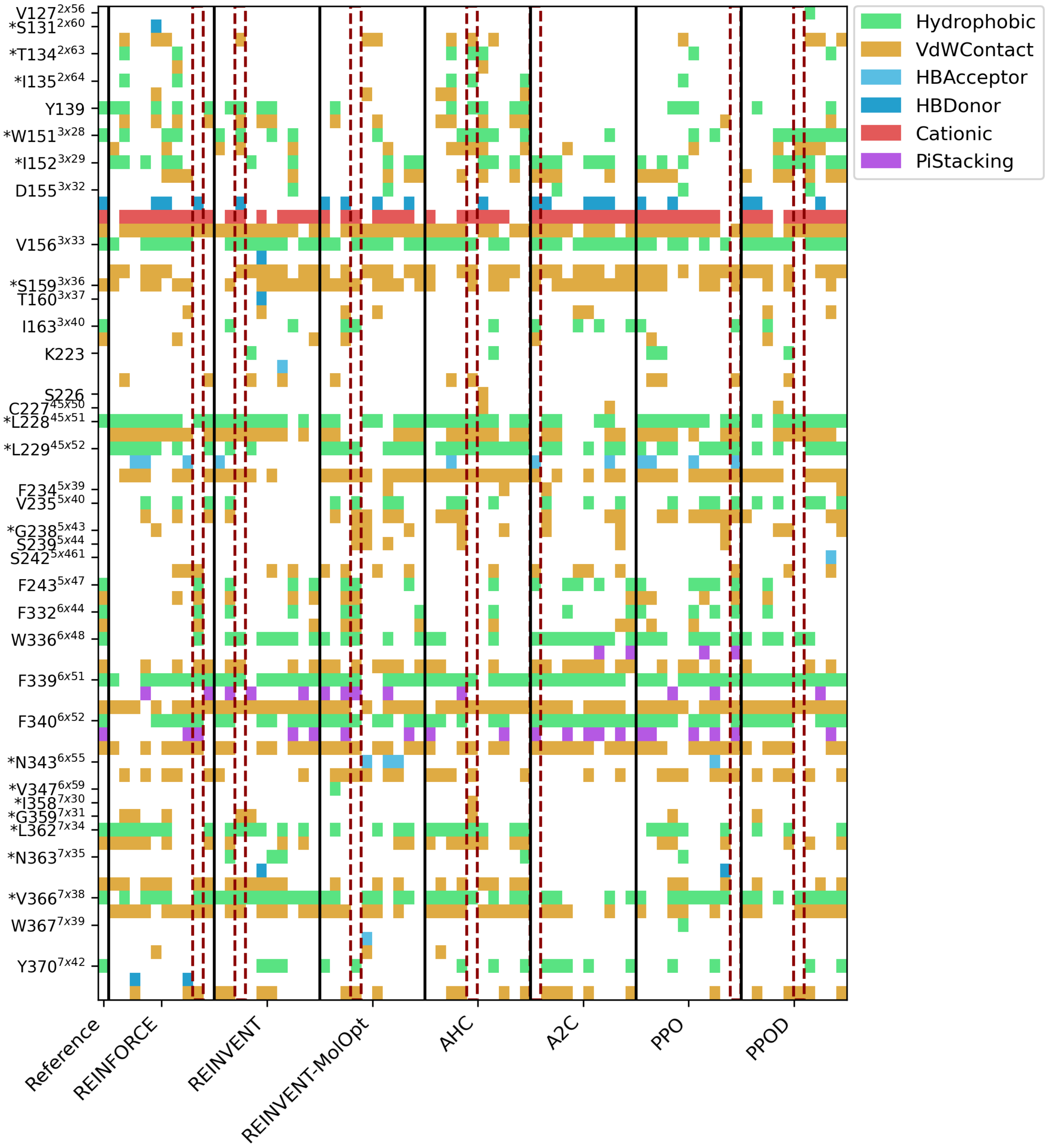

Figure S12: Protein ligand interaction fingerprints of the top 10 compounds proposed by each RL algorithm. Examples are selected for visualization of their docking poses in Figure ??. Residue labels marked with an "*" are different for the corresponding structurally aligned residue in D_2.

F. Scaffold constrained molecular generation case study

The same training dataset and pre-trained prior was used as for the MolOpt benchmark. In the first experiment, we used the objectives defined by LibINVENT[13] as re-implemented in MolScore. In the second experiment, we conducted a scaffold constrained docking experiment and took the product of three scores in the range [0-1] to define the final reward (see Table S9). The structure used was 4B05.[14] Note that during training, the inter-molecular rDock score was used as it was otherwise observed that RL would hack score optimization by improvement of intra-molecular rDock score only (data not shown). To discourage excessive exploitation, a diversity filter was used to penalize the re-generation of molecules via an 'occurance' filter i.e., up to 5 duplicates resulted in no penalty, 5-10 duplicates resulted in an increasing penalty by multiplication of value scaling linearly from 1 to 0, and more than 10 duplicates resulted in a reward of 0. Both objectives and their configuration files to reproduce them can be found at https://github.com/MorganCThomas/MolScore/.

Table S9: Description of the scoring functions to define the BACE1 scaffold constrained objective.

Scoring function	Comments	Target
5-HT_{2A} docking score (inter-molecular only)	- Substructure restraint based on bicyclic core<br>- Best score of any protonated/tautomerised/isomeric variant at pH 4<br>- Normalized based on maximum/minimum values observed at timestep	Minimize
Maximum number of consecutive rotatable bonds		< 3
Heavy atom count		< 50

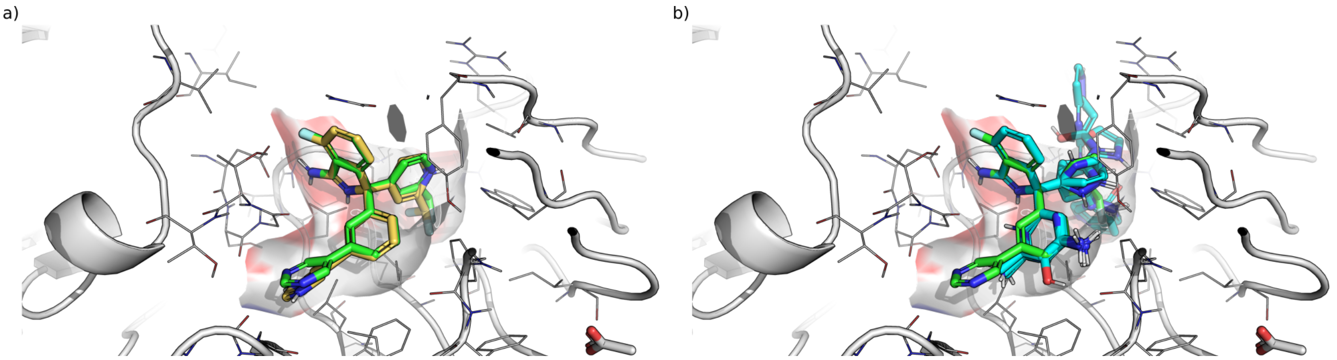

Figure S13: The docked poses of (a) AZD3839 re-docked and (b) the top 10 molecules generated by PromptSMILES with AHC. Note that the top 10 molecules often contain an secondary amine in the P1 sub-pocket to form an interaction with Tyr71 and the backbone carbonyls of Phe108 and Lys107 which interact with waters in the co-crystal structure that were removed during preparation. However, none of the top 10 molecules contain a substructure occupying the P3 sub-pocket, as the before mentioned interaction results in an orientation un-amenable to further growth into the P3 sub-pocket, although molecules outside the top 10 do show occupation of the P3 sub-pocket (data not shown).

References

(1) Bickerton, G. R.; Paolini, G. V.; Besnard, J.; Muresan, S.; Hopkins, A. L. Quantifying the chemical beauty of drugs. *Nature chemistry* **2012**, *4*, 90–98.

(2) Thomas, M.; O'Boyle, N. M.; Bender, A.; De Graaf, C. MolScore: A Scoring, Evaluation, and Benchmarking Framework for Generative Models in De Novo Drug Design. *J. Cheminf.* **2024**, *16*, 64.

(3) Guo, J.; Fialková, V.; Arango, J. D.; Margreitter, C.; Janet, J. P.; Papadopoulos, K.; Engkvist, O.; Patronov, A. Improving De Novo Molecular Design with Curriculum Learning. *Nat. Mach. Intell.* **2022**, *4*, 555–563.

(4) Polykovskiy, D.; Zhebrak, A.; Sanchez-Lengeling, B.; Golovanov, S.; Tatanov, O.; Belyaev, S.; Kurbanov, R.; Artamonov, A.; Aladinskiy, V.; Veselov, M.; others Molecular Sets (MOSES): A Benchmarking Platform for Molecular Generation Models. *Front. Pharmacol.* **2020**, *11*, 565644.

(5) Kimura, K. T.; Asada, H.; Inoue, A.; Kadji, F. M. N.; Im, D.; Mori, C.; Arakawa, T.; Hirata, K.; Nomura, Y.; Nomura, N.; others Structures of the 5-HT2A Receptor in Complex with the Antipsychotics Risperidone and Zotepine. *Nat. Struct. Mol. Biol.* **2019**, *26*, 121–128.

(6) Wang, S.; Che, T.; Levit, A.; Shoichet, B. K.; Wacker, D.; Roth, B. L. Structure of the D2 Dopamine Receptor Bound to the Atypical Antipsychotic Drug Risperidone. *Nature* **2018**, *555*, 269–273.

(7) McInnes, L.; Healy, J.; Melville, J. UMAP: Uniform Manifold Approximation and Projection for Dimension Reduction. 2018.

(8) Sauer, W. H.; Schwarz, M. K. Molecular Shape Diversity of Combinatorial Libraries: A Prerequisite for Broad Bioactivity. *J. Chem. Inf. Comput. Sci.* **2003**, *43*, 987–1003.

(9) Bouysset, C.; Fiorucci, S. ProLIF: A Library to Encode Molecular Interactions as Fingerprints. *J. Cheminf.* **2021**, *13*, 1–9.

(10) Fan, L.; Tan, L.; Chen, Z.; Qi, J.; Nie, F.; Luo, Z.; Cheng, J.; Wang, S. Haloperidol-Bound D2 Dopamine Receptor Structure Inspired the Discovery of Subtype-Selective Ligands. *Nat. Commun.* **2020**, *11*, 1074.

(11) Ropp, P. J.; Spiegel, J. O.; Walker, J. L.; Green, H.; Morales, G. A.; Milliken, K. A.; Ringe, J. J.; Durrant, J. D. Gypsum-DL: An Open-Source Program for Preparing Small-Molecule Libraries for Structure-Based Virtual Screening. *J. Cheminf.* **2019**, *11*, 1–13.

(12) Gaulton, A.; Bellis, L. J.; Bento, A. P.; Chambers, J.; Davies, M.; Hersey, A.; Light, Y.; McGlinchey, S.; Michalovich, D.; Al-Lazikani, B.; others ChEMBL: A Large-Scale Bioactivity Database for Drug Discovery. *Nucleic Acids Res.* **2012**, *40*, D1100–D1107.

(13) Fialková, V.; Zhao, J.; Papadopoulos, K.; Engkvist, O.; Bjerrum, E. J.; Kogej, T.; Patronov, A. LibINVENT: Reaction-Based Generative Scaffold Decoration for In Silico Library Design. *J. Chem. Inf. Model.* **2021**, *62*, 2046–2063.

(14) Jeppsson, F.; Eketjäll, S.; Janson, J.; Karlström, S.; Gustavsson, S.; Olsson, L.-L.; Radesäter, A.-C.; Ploeger, B.; Cebers, G.; Kolmodin, K.; others Discovery of AZD3839, a Potent and Selective BACE1 Inhibitor Clinical Candidate for the Treatment of Alzheimer Disease. *J. Biol. Chem.* **2012**, *287*, 41245–41257.
